# Developing and Recognising Undergraduate Medical Educators in a UK Medical School - A Case Study

**DOI:** 10.15694/mep.2019.000155.1

**Published:** 2019-07-23

**Authors:** Jordan Napier, Shihab E Khogali

**Affiliations:** 1 University of Dundee

**Keywords:** staff development, faculty development, Recognition of Trainers, undergraduate medical education

## Abstract

This article was migrated. The article was marked as recommended.

The UK General Medical Council (GMC) requires all UK Medical Schools to formally recognise trainers performing ‘named roles’ in undergraduate medical education. A staff development programme has been designed and implemented by the School of Medicine, University of Dundee. The programme has been made available to staff working within the School of Medicine and its partner organisations. Lessons learned through the process include, a need for: face-to-face interaction and dialogue with educators, readily available and accessible local CPD opportunities, establishment of local communities of practice, and a clear message to educators that the complexity of the ‘Recognition of Trainers’ process is at the organisational (rather than the individual) level.

## 1. Introduction and Background

Staff development has been defined as “all activities health professionals pursue to improve their knowledge, skills and behaviours as teachers and educators, leaders and managers, and researchers and scholars, in both individual and group settings” ( Steinert, 2014). A staff development programme is implemented by most organisations functioning in the continuum of medical education. These programmes are usually tailored to suit local settings and to optimise performance in a range of educational roles ( Steinert *et al*., 2016). To guide individual trainers and staff development programmes, the UK General Medical Council (GMC) adopted the 2010 Academy of Medical Educators’ (AoME) seven competency framework areas ( Table 1). As of July 2016, the GMC requires all UK Medical Schools to formally recognise, against the framework areas, all trainers performing two ‘named roles’ in undergraduate medical education: (1) ‘Lead Coordinators of Undergraduate Training’ and (2) those ‘Overseeing Students Educational Progress’ ( General Medical Council, 2012).

**Table 1.** The Academy of Medical Educator (AoME) Competency Framework Areas for Continuous Professional Development of Educators ( Academy of Medical Educators, 2010)

No.	Competency Framework Areas
**1**	Ensuring safe and effective patient care through training
**2**	Establishing an effective learning environment
**3**	Teaching and facilitating learning
**4**	Enhancing learning through assessment
**5**	Supporting and monitoring educational progress
**6**	Guiding personal and professional development
**7**	Continuing professional development as an educator

In response to the ‘Recognition of Trainers’ (RoT) requirements from the GMC, the five Scottish Medical Schools (Aberdeen, Dundee, Edinburgh, Glasgow and St Andrews) and NHS Education Scotland (NES), which organises postgraduate medical training, agreed a set of criteria for all ‘named trainers’ in Scotland - undergraduate and postgraduate. The criteria for recognition of Scottish trainers are categorised under three headings (A) Educational Governance Requirements (B) Role-specific Requirements (C) Generic Teaching Skills Requirements ( Table 2).

**Table 2.** Criteria Agreed by Scottish Medical Schools and NES (NHS Education for Scotland) for Recognition of All Scottish Trainers (Published by: Scotland Deanery, 2016)

**A**	**Educational Governance Requirements** Be currently practising within their field (for undergraduate trainers this may include academic practice or health professionals in disciplines other than medicine). Comply with all aspects of Good Medical Practice (as published by the GMC). Comply with all legal, ethical and professional obligations including completion of any mandatory training requirements. Have appropriate time allocated for their role.
**B**	**Role-specific Requirements** Demonstrate awareness of the curriculum and level of students/trainees. Demonstrate awareness of their role and how that role fits with other educational and clinical roles. Know how to get support if needed and know about the relevant education organisers’ (EOs) quality assurance (QA) procedures.
**C**	**Generic Teaching Skills Requirements** Produce evidence of ongoing development across all relevant Framework areas to demonstrate an appropriate level of teaching competence. Provide evidence of appropriate training and/or experience for their teaching role.

The joint Scottish Medical Schools/NES criteria within the ‘Generic Teaching Skills Requirements’ “aims to ensure that all trainers have attained a basic, minimum level of competence and that they continue to develop their skills as appropriate for their role” ( Scotland Deanery, 2016). Initial and ongoing RoT are built into the existing appraisal processes. All educators, including those who do not hold a ‘named trainer role’, are encouraged to include their teaching role in appraisal processes ( Scotland Deanery, 2016). Educators who hold a ‘named trainer’ role have been allowed flexibility to demonstrate how the RoT requirements are met for both initial and ongoing recognition. Three routes have been identified for initial RoT in Scotland: (1) Completion of an accredited medical education course (2) Full Membership/Fellowship of the Academy of Medical Educators (AoME) or Fellowship of the Higher Education Academy (3) Presentation of a portfolio of evidence mapped against the seven competency framework areas ( Scotland Deanery, 2016).

In this paper we describe approaches adopted by the School of Medicine, University of Dundee for the development and support of undergraduate medical educators. It is hoped that the paper and the lessons learned, from the local implementation of the RoT process, will be of interest to those engaged in the development of health professions educators in general.

## 2. Approaches to Developing and Recognising ‘Named’ Undergraduate Trainers at the School of Medicine, University of Dundee

### 2.1 Timeline for the Implementation of RoT Requirements

Table 3 summarises key national milestones and developments in relation to RoT, and how the School of Medicine, University of Dundee responded to these.

**Table 3.** Timeline for the Implementation of ‘Recognition of Trainers’ (RoT) Requirements at the School of Medicine, University of Dundee

Time period	Key milestones and actions taken
2012	The UK General Medical Council (GMC) published the implementation plan for RoT.
2012 - onwards	The School of Medicine, University of Dundee initiated a process to continuously identify roles which require formal recognition. Information about RoT requirements has been disseminated, offering support and guidance to those requiring it. This includes face-to-face communication utilising established fora such as the local monthly ‘Teaching Leads’ meeting. Six ‘Core Skills’ workshops, mapped to the seven competency framework areas, have been designed and implemented by the School of Medicine, University of Dundee.
2015	Scottish Trainer Framework website went live. The School of Medicine, University of Dundee designed an additional workshop entitled ‘Building and Maintaining Your Trainer Portfolio’.
2016	Deadline for providing the GMC with a list of recognised trainers from all UK Medical Schools.
2017 - onwards	Current and prospective ‘named trainers’ are given the opportunity to attend the workshop on ‘Building and Maintaining Your Trainer Portfolio’. All staff teaching in the Dundee undergraduate medical programme are given the opportunity to attend any or all of the six ‘Core Skills’ workshops. All staff development activities coordinated through the School of Medicine’s are mapped to the competency framework areas, and marketed as such. Certificates of attendance are provided with the relevant framework areas.

### 2.2 Undergraduate ‘Named Trainer’ Roles at the School of Medicine, University of Dundee

At the time of writing this paper, the School of Medicine, University of Dundee has 134 undergraduate ‘named trainer’ roles that require formal recognition ( Table 4). These roles are held by a total of 129 members of University or Honorary University staff, working within the National Health Service (NHS).

**Table 4.** Current ‘Named Trainer’ Roles at the School of Medicine, University Dundee

Role category	Number of roles
‘Lead Coordinators of Undergraduate Training’	118
‘Overseeing Students Educational Progress’	16

The majority of staff holding ‘named trainer’ roles within the Dundee undergraduate medical programme (88%, 114 out of 129) needed to present a portfolio of evidence (mapped against the seven competency framework areas) to gain initial recognition. About 12% (15 out of 129) gained initial recognition by holding a recognised qualification and/or by membership of a recognised educational body.

### 2.3 Designing and Implementing Staff Development Opportunities to SupportUndergraduate ‘Named Trainers’

#### 2.3.1 ‘Core Skills’ Workshops Mapped to the Competency Framework Areas

A set of six half-day ‘Core Skills’ workshops have been designed and implemented by the School of Medicine, University of Dundee ( Table 5). The workshops have been mapped to the competency framework areas and made available to all staff teaching in the Dundee undergraduate medical programme. Trainers are allowed flexibility (according to their needs) to participate in any (or all) of the six workshops. The participants can attend a standalone half-day workshop, multiple half day workshops over a period of time, or all six ‘Core Skills’ workshops as an intensive three-and-a-half-day programme.

**Table 5.** Core Skills Workshops. A Programme of Workshops, Mapped to the Competency Framework Areas, as Developed by the School of Medicine, University of Dundee

Workshop Title	Learning Objectives	Competency Framework Areas Covered
**Core Skills 1: Planning for learning**	Explain what is meant by ‘learning needs’ and describe how they can be identified Use action verbs to write good learning objectives List the elements of a good ‘lesson plan’ and explain how they relate to each other Consider ways of improving ‘unplanned’ or opportunistic teaching through more effective planning Apply basic evaluation skills to improve teaching	Area 2: Establishing an effective learning environment Area 3: Teaching and facilitating learning Area 7: CPD as an educator
**Core Skills 2: Small group teaching**	Explain the benefits of teaching in small groups Describe the role of the tutor within the group Identify skills needed to facilitate a small group session effectively Select from a range of methods available to encourage active participation of learners Plan a small group session to meet specified learning needs or outcomes	Area 2: Establishing an effective learning environment Area 3: Teaching and facilitating learning Area 7: CPD as an educator
**Core Skills 3: Presentation skills**	Explain why lectures are still an important part of the teaching toolkit Describe the role of the lecturer and identify skills required by an effective lecturer List some of the common problems encountered when using audiovisual aids Describe how technology can enhance a presentation and encourage active participation of learners Use a simple planning model to structure a lecture or presentation to meet specified learning needs or outcomes	Area 2: Establishing an effective learning environment Area 3: Teaching and facilitating learning Area 7: CPD as an educator
**Core Skills 4: Clinical teaching**	Explain the importance of role modelling Apply the four-step model for skills-teaching Plan your teaching with patients more effectively Describe two techniques that can support delivery of ‘unplanned’ teaching encounters Give consideration to improving patient care when selecting patients for teaching	Area 1: Ensuring safe and effective patient care through training Area 2: Establishing an effective learning environment Area 3: Teaching and facilitating learning Area 7: CPD as an educator
**Core Skills 5: Assessment**	Explain the difference between formative and summative assessment Define ‘assessment to a standard’ Describe some of the tools commonly used in medical assessment Decide whether an assessment tool is valid, reliable and practical Choose an appropriate assessment tool Explain how to minimise bias when conducting assessments	Area 3: Teaching and facilitating learning Area 4: Enhancing learning through assessment Area 7: CPD as an educator
**Core Skills 6: Supporting learning**	Describe the various aspects making up the learning environment, including both physical and psychological elements Consider the learner in context and take account of external factors which may enhance or hinder learning Identify traits of learners in difficulty and consider both formal and informal support mechanisms Use a simple tool to give structured feedback	Area 2: Establishing an effective learning environment Area 3: Teaching and facilitating learning Area 5: Supporting and monitoring progress Area 6: Guiding personal and professional development Area 7: CPD as an educator

#### 2.3.2 A Workshop to Support Building and Maintaining a Trainer Portfolio

A workshop entitled ‘Building and Maintaining Your Trainer Portfolio’ has been designed, implemented and evaluated. This workshop has been made available to those undertaking a recognised trainer role and also those who wish to take on such roles in the future. Participants in this workshop are encouraged to engage in discussions and ask questions. Teaching strategies adopted during this workshop include, small group teaching techniques such as buzz groups and snowballing ( McCrorie, 2013), and the use of case studies. During the workshop, participants are presented with a ‘dummy’ educator portfolio to critique and then map each piece of evidence to the competency framework areas. As part of the workshop, participants are signposted to local staff development opportunities (including, the six ‘Core Skills’ workshops) and how to access these. Participants are also made aware that all staff development opportunities planned and promoted by the School of Medicine, University of Dundee are mapped to the competency framework areas, to make it simple to plan CPD and fulfil the requirements for RoT.

Between June 2017 and May 2018, the ‘Building and Maintaining Your Trainer Portfolio’ workshop has been attended by a total of 44 participants, of different backgrounds. Attendance at each workshop ranged from 5 to 15 participants. The workshop has been evaluated, as part of routine evaluation of staff development programmes at the School of Medicine, University of Dundee. The evaluation questionnaire has been completed by 31 participants (70% response rate). Results showed that participants ranked the workshop highly in achieving its learning objectives ( Table 6).

**Table 6.** Participants Feedback from the Workshop on ‘Building and Maintaining Your Trainer Portfolio’ (n = 31)

For each learning objective below, rank your ability to:	Question unanswered	Likert-like scale, where: 1 = poor/low, 3= average, 5= excellent/high				
		1	2	3	4	5
**Explain how ‘named trainers’ will be expected to collate a portfolio**	1 (3%)	0	0	0	13 (42%)	17 (55%)
**Describe the purpose of an educator portfolio in terms of quantity, quality and engagement**	0	0	0	0	16 (52%)	15 (48%)
**Evaluate the quality of supporting information**	0	0	0	1 (3%)	12 (39%)	18 (58%)
**Identify some pieces of evidence you could supply**	0	0	0	0	15 (48%)	16 (52%)

Participants commented that the strengths of the workshop have been in its informality and in the opportunity to ask questions openly. They also commented on the value of the practical tips shared during the workshop, and on the opportunity to discuss the RoT process with peers (with one participant commenting that the workshop had ‘ *taken the mystery out of the process*’). Some participants commented on the lack of clarity around the volume of evidence needed to meet the required standards. One participant commented ‘it’s still difficult to grasp what quality the evidence should be’. These comments reflect the flexibility of the RoT process, but also the lack of clear guidance for trainers. Unresolved questions after the workshop included; ‘how often should evidence be collected?’ and ‘What is the volume of evidence required?’

#### 2.3.3 Developing and Publishing a ‘Getting Started’ Booklet on Trainer Recognition

A booklet entitled ‘Getting Started with Trainer Recognition’ has been developed by School of Medicine’s Staff Development team. All educators at the School of Medicine, University of Dundee are given online access to the booklet. Participants on the trainer portfolio workshop are given a hard copy of the booklet, which includes sections that they can complete within the workshop to start evidencing their engagement, experience and development regarding their educational practice. This allows participants to leave the session having made a start on collating evidence for their portfolio.

#### 2.3.4 Arranging for One-to-one Support and for Peer Observation of Teaching

Access to one-to-one support is made available to all ‘named trainers’ in the Dundee undergraduate medical programme, and also any teachers who may wish to take on a role as a ‘named trainer’ in the future. All educators in the Dundee undergraduate medical programme are also encouraged to consider arranging for peer-observation of teaching.

### 2.4 Developing Staff Beyond Generic Teaching Skills

To provide a more advanced level for staff development, beyond generic teaching skills, we established a series of masterclasses entitled ‘Medical Education Masterclasses: Sharing the Good Stuff’. These provided opportunities to share good educational practice. Presenters at the masterclasses come from a range of backgrounds including, for example, Medical Education, Learning Technology, Health Professions’ Practice Development, Education and Cognitive Psychology. All staff at the School of Medicine are encouraged to attend and participate in the masterclasses, which usually take the format of interactive workshops. Examples of Masterclass titles and background of presenters are shown in Table 7.

**Table 7.** Examples of Medical Education Masterclasses Recently Delivered at Dundee School of Medicine, University of Dundee

Masterclass Title	Background of Presenter(s)	Home Institution of Presenter(s)
The Use of Storytelling in Medical Education	Medical Education	University of Dundee
The Value of Blogging for Medical Educators	Medical Education; Learning Technology	University of Dundee
Flourishing Workplaces	Health Professions’ Practice Development	Queen Margaret University, Edinburgh
The Scholarship of Teaching and Learning	Education	University of Dundee
Writing A Journal Article	Medical Education	University of Dundee
Epistemology in Medical Education	Education	University of Aberdeen
Exploring Feedback in Context at Medical School	Medical Education	University of Dundee
Lessons from Cognitive Psychology for Higher Education	Cognitive Psychology	University of Dundee

To make the masterclasses more attractive, they are mapped to the competency framework areas ( Table 8) hence allowing staff opportunities to use these as evidence in educational portfolios.

**Table 8.** Mapping of the Medical Education Masterclasses to the Competency Framework Areas for RoT

Masterclass Title	Competency Framework areas the masterclass maps to:
The Use of Storytelling in Medical Education	Area 3: Teaching and facilitating learning Area 7. CPD as an educator
The Value of Blogging for Medical Educators	Area 2: Establishing an effective learning environment Area 3: Teaching and facilitating learning Area 6: Guiding personal and professional development Area 7. CPD as an educator
Flourishing Workplaces	Area 1: Ensuring safe and effective patient care through training Area 2: Establishing an effective learning environment Area 7. CPD as an educator
The Scholarship of Teaching and Learning	Area 7. CPD as an educator
Writing A Journal Article	Area 7. CPD as an educator
Epistemology in Medical Education	Area 3: Teaching and facilitating learning Area 7. CPD as an educator
Exploring Feedback in Context at Medical School	Area 2: Establishing an effective learning environment Area 3: Teaching and facilitating learning Area 4: Enhancing learning through assessment Area 5: Supporting and monitoring progress Area 7. CPD as an educator
Lessons from Cognitive Psychology for Higher Education	Area 3: Teaching and facilitating learning Area 4: Enhancing learning through assessment

## 3. Discussion

The majority of undergraduate medical educators are practicing healthcare professionals, who received no formal training in education. Hence, it is important to support healthcare educators to skilfully transition between their specialist and educational roles ( Lazzari, Martini and de Amorim Busana, 2015). The RoT process has been introduced by the GMC as a measure to “improve the standard of medical training” ( Rimmer, 2015). However, it has caused mixed emotional responses, with some trainers reporting feeling demoralised and demotivated ( Crichton and Jones, 2018). In this article, we described approaches adopted by the School of Medicine, University of Dundee to support undergraduate medical educators to fulfil the RoT requirements.

We adopted a strategy of motivating trainers to access readily available CPD opportunities for the purpose of improving their teaching practice, rather than simply ticking boxes to meet the required standards. In the context of a teaching hospital, all doctors are expected to teach. This can act as a barrier to engagement in educational development, and promote a box ticking culture. Cheesman (2009) described such a situation as an “imposition” and feared that teaching may become a tick box exercise rather than something enjoyable for doctors. The availability of structured CPD opportunities may also help support educators when completing a reflective portfolio; a process which may be viewed by some as a time-consuming and meaningless exercise ( Heeneman *et al*., 2019). Through the Medical Education Masterclass series, we attempted to move away from purely training educators in the skills required for teaching, to opening up conversations about what it means to be a medical educator. This may also help with the development of “teachers’ identities in addition to their skills” ( Steinert, O’Sullivan and Irby, 2019)

Our experiences suggested that, face-to-face interaction and dialogue with medical educators made it easier for them to accept and comprehend the introduction of the RoT process. These experiences highlighted a need for personalising communications, at the local level, about a regulator-imposed mandatory change. Such changes are usually introduced by face-less organisations, making them difficult and complex to communicate at the local level, including why, what, when, and how. Hence, facilitating such a change may not be achievable through written and online communications alone.

Our journey has also highlighted a need for informal faculty development opportunities, including the need for establishment of local communities of practice. This is in agreement with the suggestions by Steinert *et al*., (2016) “that belonging to a community can enhance a sense of membership, promote transfer of training, and foster the attainment of individual goals”. Through this, we hope to achieve a change in culture that benefits the School of Medicine and partner organisations. Moving forward our goals for developing and recognising undergraduate educators, include creating spaces for and facilitating the formation and growth of communities of practice. As part of this, we encouraged the development of a ‘Dundee Discover MedEd Blog’ ( https://sites.dundee.ac.uk/discovermeded/ - accessed: 17th July 2019) that aims to share scholarly medical education work done at the School of Medicine, University of Dundee. We see the need for the fostering of these communities and spaces as key to enabling medical educators to flourish.

In order for the RoT process, and approaches adopted locally, and the emphasis on educational CPD to be truly valuable, we need to be able to see the transfer of improved teaching practice into the learning environment. The School of Medicine, University of Dundee is currently conducting ongoing research in collaboration with other European institutions with the aim of understanding how learning from faculty development programs is transferred into the clinical learning environment.

## Take Home Messages

The following may help facilitate the development of undergraduate medical educators:

• Face-to-face interaction and dialogue with educators, including effective communication about regulator-imposed changes.
• Readily available and accessible local CPD opportunities, mapped to an identifiable framework.
• Establishment of local communities of practice.

## Notes On Contributors

Miss Jordan Napier is Staff Development Officer, leading the Faculty Development programme for staff involved in teaching medical students at the School of Medicine, University of Dundee.

Dr Shihab Khogali is a Clinical Senior Lecturer in Medical Education at the School of Medicine, University of Dundee. He currently acts as medical education scholarship liaison lead, linking the undergraduate medical curriculum to the Centre for Medical Education, the wider School of Medicine and the wider University. He conceived the idea of Medical Education Masterclasses and currently co-leads these. He also leads a range of educational programmes within the undergraduate medical curriculum, including: the cardiovascular course, the physiology theme, the introduction to curriculum outcomes course, and a transition course into clinical years.

## Declarations

The author has declared that there are no conflicts of interest.

## Ethics Statement

The Chair of the School of Medicine, University of Dundee Ethics Committee has indicated that formal approval was not required for the feedback collected, as part of routine evaluation of staff development programmes, from the workshop on ‘Building and Maintaining Your Trainer Portfolio’.

## External Funding

This article has not had any External Funding
