# Supplementary material for: Developing and Recognising Undergraduate Medical Educators in a UK Medical School - A Case Study
Source: MedEdPublish (2016). 2019 Jul 23;8:155. [Version 1] doi: 10.15694/mep.2019.000155.1 (PMC10712515; doi:10.15694/mep.2019.000155.1)
Supplement: Peer review history

# Reviewer report: Reviewer response for version 1

**DOI:** 10.21956/mep.19899.r30122

**Published:** 2019-10-17

**Authors:** Trevor Gibbs

**Affiliations:** 1 AMEE

This review has been migrated. The reviewer awarded 4 stars out of 5 As someone actively involved in faculty development, I enjoyed reading this very informative paper, which clearly outlines the development in the UK - perhaps a model that can be copied by other countries. I was pleased to see that there was mention of the need to recognise educators as scholars in their own practice, but would have liked to have seen more discussion regarding scholarly recognition, promotional issues related to having ticked all the boxes and more regarding the self esteem and self actualisation that should be afforded to our educators. Some I would presume these to be the softer measurements, but I do believe they are very important.An important paper for all faculty developers to read

Reviewer Expertise:

NA

No decision status is available

# Reviewer report: Reviewer response for version 1

**DOI:** 10.21956/mep.19899.r30120

**Published:** 2019-07-24

**Authors:** BALAJI ARUMUGAM

**Affiliations:** 1 TAGORE MEDICAL COLLEGE AND HOSPITAL

This review has been migrated. The reviewer awarded 4 stars out of 5 Thanks for the invite to review this article. This is excellent case study article which speaks about the competency framework for the educators, named roles of the educators, workshops to map the competency frameworks and their feedback. The two named roles of the educators areLead Coordinators of Undergraduate Training’‘Overseeing Students Educational Progress......were clearly explained in the text. Its wonderful to know that the training workshops on sex core skills, with SLOs, covering the entire competency framework areas for the educators was programmed and implemented with the time frame also. This model of RoT article will really be useful for all the medical educators those who are working to improve the quality of medical education in their respective places. Good work and congrats to the authors.

Reviewer Expertise:

NA

No decision status is available

# Reviewer report: Reviewer response for version 1

**DOI:** 10.21956/mep.19899.r30121

**Published:** 2019-07-23

**Authors:** Felix Silwimba

**Affiliations:** 1 University of Lusaka

This review has been migrated. The reviewer awarded 5 stars out of 5 this is an excellent case study in medical education. I recommend its adoption in Low Middle income countries . to strengthen medical education.

Reviewer Expertise:

NA

No decision status is available
